# My 40-Year History with *Cronobacter/Enterobacter sakazakii* – Lessons Learned, Myths Debunked, and Recommendations

**DOI:** 10.3389/fped.2015.00084

**Published:** 2015-11-27

**Authors:** John J. Farmer

**Affiliations:** ^1^Formerly affiliated with United States Public Health Service, Atlanta, GA, USA (Retired); ^2^Silver Hill Associates, Stone Mountain, GA, USA

**Keywords:** *Cronobacter*, *Enterobacter sakazakii*, powdered infant formula, safety, neonatal meningitis, risk assessment, government regulations, recommendations

## Abstract

Much has been learned about organism in the *Cronobacter/Enterobacter sakazakii* complex since I first named and described *Enterobacter sakazakii* in 1980. However, there are still wide knowledge gaps. One of the most serious is that are still many uncertainties associated with assessing the public health risk posed by these bacteria, particularly in neonatal meningitis. Over the last few decades, *Cronobacter* contamination of commercial powdered infant formula products has apparently been reduced, but it is still an ongoing problem. The powdered infant formula industry still cannot produce powdered formula that is free of bacterial contamination with *Cronobacter*, other *Enterobacteriaceae*, other pathogenic bacteria, and other microorganisms. Until this happens, infants and other will be at risk of becoming infected when they ingest contaminated formula.

## Introduction

*“Those who don’t know history are destined to repeat it.”* – Edmund Burke (1729–1797, British Statesman and Philosopher). In this review, I look at this familiar quotation in the context of my 40+ year-history with *Cronobacter – Enterobacter sakazakii* plus its sibling organisms that are now classified in the genus *Cronobacter*. There are now over 600 papers about this genus of *Enterobacteriaceae*; however, there are many myths and some knowledge gaps. There are also misconceptions and statements being made that simply are not supported by fact. Many of these are based on the lack of knowledge about the organism’s early history and the first few decades of research that laid the foundation for our current knowledge. The purpose of this review is to debunk, correct, clarify, and recommend. It is based on my long history and many different experiences with “Esak.”

*Cronobacter* researcher Stephen J. Forsythe wrote a paper in 2012 with an intriguing title – “Myths and legends of *Cronobacter* – a new bacterial pathogen of babies?” (1). In this short paper, he listed and described seven items that he called “myths.” I really like his idea and wish to expand the discussion he started. Each of my discussion items will be in the form of one of the followings: observation, fact, question, myth, or recommendation.

## History

*Facts:* The first documented *Cronobacter* isolate dates back only to 1950. The first strain isolated from a human clinical specimen was in 1953. The first documented case of neonatal meningitis was in 1958 (see below).

The recorded history for *Cronobacter* organisms is short but they have certainly existed for millions of years (tens of millions of years? hundreds of millions of years?). It is only within the last few years that we have understood the complexity of the group and begun to use precise and accurate names in describing the species, subspecies, biogroups, and other subgroups, such as ribotypes, PFGE pattern types, and sequence types.

*1890’s to the 1920’s*: The bacteriological era was in its infancy. Bacteria were being described and named but with incomplete and imprecise descriptions. Many yellow-pigmented bacteria were described, given names, and classified in many different genera. A partial list includes: “coliform,” “yellow-pigmented coliform bacteria,” “pigmented cloacae A,” “*Serratia* species,” “*Enterobacter* species,” “*Erwinia* species,” “*Chromobacterium typhiflavum*,” “*Chromobacterium* species,” “unidentified *Enterobacteriaceae*,” and perhaps other names. The term “yellow pigmented coliform” was a vernacular term that appeared in the literature. It would include strains of *Enterobacteriaceae* that produce a yellow pigment and fermented lactose and produced gas during this fermentation. An organism described as a “yellow pigmented coliform” could be *E. sakazakii* or could be one of the other species or biogroups of *Enterobacteriaceae* that share these few phenotypic properties. When a culture with one of the above names exits in a culture collection, it can be tested with current methods to determine its correct identification and name. This was done with the cultures deposited in England’s National Collection of Type Cultures (NCTC) and several were determined to be in the *Enterobacter sakazakii* complex as described in Farmer et al. (2). Unless cultures were preserved, it is impossible to determine their correct identification based on today’s knowledge.

*1929*: A case of septicemia due to a “pigmented coliform” was described by Pangalos (3) who proposed the name *Bacillus rubroluteus*. However, some of the characteristics of this bacterium do not fit those of *E. sakazakii*: growth in broth was uniformly turbid (“Le bacille pousse sur bouillon en donnant un trouble homogené.”) in contrast to the growth of *E. sakazakii*, which characteristically settles to the bottom, leaving the upper layer clear; the pigment was yellow-brick rather than yellow “like *Bacterium flavum*.” The isolate was from a woman suffering from a febrile infection after curettage; not from a newborn. The organism was later renamed *Serratia rubroluteum*. No cultures of *Serratia rubroluteum* have apparently survived, so it is not possible to determine its true identity based on current knowledge.

*1950*: A strain of *Enterobacteriaceae* was isolated from a “tin of dried milk” and was sent to England’s National Collection of Type Cultures (NCTC). There it was given the designation NCTC 8155. It was re-characterized in the late 1970’s and identified as *E. sakazakii* biogroup 1 (2). This is the earliest date for a strain of this organism and is also the first documented isolate of *Cronobacter* from food and from a “dried milk” type of product. In 2011, Joseph and Forsyth (4) reported additional results for this strain, which they had obtained and then studied about 60 years after it was first isolated. They determined that is was “*Cronobacter sakazakii* sequence type 4,” which they found to be “a highly stable clone with a high propensity for neonatal meningitis.”

*1953*: A strain from abdominal pus was submitted to NCTC and given the designation NCTC 9238. It was later identified as *E. sakazakii* biogroup 1 (2). This is the earliest date of an isolate of *Cronobacter* from a human clinical specimen.

*1954*: A strain from water was submitted to NCTC and given the designation NCTC 9529. This isolate came from the Metropolitan Water Board. It is most likely was not isolated from drinking (potable) water, but may have come from the river water entering the treatment works rather than post-treatment (Barry Holmes, Director of NCTC, personal communication). It was identified as *E. sakazakii*, and then re-studied later. According to Joseph et al. (5) and to http://bacteria.ensembl.org/cronobacter_universalis_nctc_9529/Info/Annotation/# about this organism’s correct identification is *Cronobacter universalis*. This is the first documented water/environmental isolate of *Cronobacter*.

*1958*: This is the first documented neonatal meningitis case and the first documented outbreak (2 cases) due to *E. sakazakii*. It occurred at Osterhills Hospital (St. Albans City Hospital), England (6). The organism was identified as a “pigmented coliform bacterium.” Fortunately, these isolates were sent to M. T. Parker, of the Manchester (England) Public Health Laboratory who studied and preserved them.

*1961*: Urmenyi and White-Franklin publish their report about the Osterhills Hospital meningitis cases in *The Lancet* and used the term “pigmented coliform” in the title of the manuscript (6).

*1965*: The second documented case of meningitis due to *E. sakazakii* occurred at the city and county hospital of Odense, Denmark (7). This isolate was compared by M. T. Parker to the 1958 Osterhills Hospital isolates, and it was almost identical to the strains isolated in 1958. These organisms were again identified as a yellow-pigmented *Enterobacter cloacae* rather than as *Enterobacter sakazakii*.

*1972 to 1976*: DNA–DNA hybridization experiments showed that *Enterobacter cloacae* and yellow-pigmented *E. cloacae* were closely related, but the genetic relationship was not close enough to be classified as belonging to the same species (8, 9).

*1977*: The name *Enterobacter sakazakii* first appeared in print. This was in a widely distributed CDC publication (10). I also used this name in an oral presentation at the 1977 American Society for Microbiology national meeting.

*1977–79*: The name *Enterobacter sakazakii* was not validly published under the rules of the *Bacteriological Code* and should have appeared in parentheses and written as “*Enterobacter sakazakii*.”

*1980*: The name *Enterobacter sakazakii* was “officially published” according to the rules of the *Bacteriological Code*, and thus the name became a validly published species that can be written as – *Enterobacter sakazakii*, Farmer et al. (2). The species description included 15 subgroups, which were classified and named as 15 distinct biogroups. Biogroup 15 was very different in its biochemical properties from the other 14 biogroups. We speculated that this unusual biogroup might eventually be classified as a separate species, which indeed it was, 27 years later (11).

*1980 onward*: Once *Enterobacter sakazakii* was named and described, case reports of *E. sakazakii* neonatal meningitis and other papers about it began to appear in the literature.

*1980 to 2007*: From 1980 to 2007, *Enterobacter sakazakii* was considered to be a single well-defined bacterial species with 15 biogroups as named and described in the original paper (2). During this time, commercial and other identification systems and methods gave an identification of *Enterobacter sakazakii* (or *Enterobacter cloacae)*. Each of these identifications needs to be reevaluated based on the establishment of *Cronobacter* in 2007 (see below).

*1983*: Muytjens et al. (12) summarize eight neonatal meningitis and sepsis cases of *Enterobacter sakazakii* from The Netherlands. This was the first large series of neonatal infections to be reported and the first outbreak with a thorough analysis. The cases were over a 4-year time period (1977–81) and comprised all the *Enterobacter sakazakii* cases detected in the entire country.

*1987*: Muytjens et al. (13) studied the contamination rate of commercial powdered formula products produced in 35 different countries. They found *E. sakazakii* and other *Enterobacteriaceae* were common contaminants. *E. sakazakii* was detected in powdered formula made in 13 different countries. This paper really marked the beginning of the association of *E. sakazakii* with powdered formula. This association and the danger of contaminated powdered formula was emphasized in an editorial by Muytjens and Kollée (14).

*1988*: This is the date of the first documented *E. sakazakii* outbreak in the United States (15). There were two bacteremia cases and two additional cases of colonization in a neonatal intensive care unit. All four infants had been fed a powdered protein hydrolyzate formula that had been mixed in a blender. The blender and an open can of the powdered protein hydrolyzate formula were cultured and found to be contaminated with *Enterobacter sakazakii*. This raised the question that does not have an answer – did the powdered formula contain *Enterobacter sakazakii* and contaminate the blender, or did the contaminated blender contaminate the formula?

*2001*: There was an outbreak of *Enterobacter sakazakii* at a Tennessee hospital with one case of meningitis and eight additional cases with *Enterobacter sakazakii* colonization. It was caused when infants were fed Portagen, a commercial powdered formula produced by Mead Johnson. This outbreak marked the beginning of the U. S. Food and Drug Administration’s interest in contamination of powdered infant formula products and especially contamination with *Enterobacter sakazakii*.

*2002, April 11*: In response to the Portagen outbreak, the U. S. Food and Drug Administration issued a letter to American health community: “Health Professionals Letter on *Enterobacter sakazakii* Infections Associated with Use of Powdered (Dry) Infant Formulas in Neonatal Intensive Care Units.” (16)

*2002, July 26*: In response to the Portagen outbreak, the U. S. Food and Drug Administration began a program to determine the degree of *E. sakazakii* contamination of powdered infant formula products (and associated raw ingredients) made by American companies (17). This FDA study determined that 5 of 22 (22.7%) of the batched samples of “finished product” were contaminated with *E. sakazakii* (18). Two of 69 samples of raw ingredients were contaminated with *E. sakazakii* (18).

*2003, March 18–19*: In further responses to the Portagen outbreak, the Food Advisory Committee of the U. S. Food and Drug Administration held a meeting of experts to discuss *E. sakazakii* infections in babies and infants with emphasis on contamination of powdered infant formula made by U. S. companies. At the meeting Dr. Don Zink presented the results from the FDA sampling and testing for *E. sakazakii* described above.

Ref (19) gives the complete set of slides for all eight presentations that were given.

*2004*: The World Health Organization published the first of three books on *Cronobacter/Enterobacter sakazakii* (20).

*2006*: The World Health Organization published the second of three books on *Cronobacter/Enterobacter sakazakii* (21).

*2007*: Cronobacter was proposed as a new genus to include the organisms formerly classified as *Enterobacter sakazakii*. *Cronobacter* Iverson et al. – had eight different organisms including four named species, one unnamed species, and five named subspecies:

Cronobacter sakazakii
  *Cronobacter sakazakii* subspecies *sakazakii*
  *Cronobacter sakazakii* subspecies *malonaticus*
Cronobacter dublinensis
  *Cronobacter dublinensis* subspecies *dublinensis*
  *Cronobacter dublinensis* subspecies *lactaridi*
  *Cronobacter dublinensis* subspecies *lausanensis*
Cronobacter muytjensii
Cronobacter turicensis
*Cronobacter* genomospecies 1 (a distinct species, but unnamed)

*2008*: The World Health Organization published the third of three books on *Cronobacter/Enterobacter sakazakii* (22). The book *Enterobacter sakazakii* (23, 24) was published by the American Society for Microbiology in its series *Emerging Issues in Food Safety*.

*2009, January 22–23*: The First International Conference on *Cronobacter (Enterobacter sakazakii*) was held at O’Reilly Hall, University College, Dublin, Ireland^1^.

At this conference Dr. Angelica Lehner reported that *Enterobacter sakazakii* can be in a viable but non-culturable state and thus escape detection when only “normal” microbiological culturing methods are used.

*2012–13*: Three new species of *Cronobacter* were described – *Cronobacter universalis*, *Cronobacter pulveris*, and *Cronobacter zurichensis*.

*2015*: Ten species and three subspecies of *Cronobacter* have now been named, described, and have standing in nomenclature:

*Cronobacter* Iversen et al. (25)
  *Cronobacter condimenti* Joseph et al. (5)
  *Cronobacter dublinensis* Iversen et al. (25)
  *Cronobacter dublinensis* subsp. *dublinensis* Iversen et al. (25)
  *Cronobacter dublinensis* subsp. *lactaridi* Iversen et al. (25)
  *Cronobacter dublinensis* subsp. *lausannensis* Iversen et al. (25)
*Cronobacter helveticus* Brady et al. (26)
  *Cronobacter malonaticus* Iversen et al. (25)
  *Cronobacter* muytjensii Iversen et al.
  *Cronobacter pulveris* Brady et al. (26)
  *Cronobacter sakazakii* (2, 25)
  *Cronobacter turicensis* Iversen et al. (25)
  *Cronobacter universalis* Joseph et al. (5)
  *Cronobacter zurichensis* Brady et al. (26)

See http://www.bacterio.net/cronobacter.html and http://www.bacterio.net/-allnamesac.html for complete details for the above organisms and as a convenient way to find updates on new organisms in this genus and new organisms in other genera. Additional *Cronobacter* species probably exist but await discovery and description.

## Nomenclature and Classification

*Two myths*: *Enterobacter sakazakii* had been moved to a new genus *Cronobacter*. All strains originally known as *Enterobacter sakazakii* are now *Cronobacter sakazakii*.

Most scientists are not familiar with the *Bacteriological Code* whose principals and rules govern the naming and classifying bacteria. Accurate statements in relation to the myths above are:

(1) The original name and classification of this group of organisms now known as *Cronobacter* is “*Enterobacter cloacae* – yellow pigmented strains.” An even older name is “pigmented coliform.” However, most strains given the name “pigmented coliform” would probably not be *Cronobacter* if they could be re-studied and precisely identified.
(2) The “first proposed reclassification” was by Farmer et al. who named and described *Enterobacter sakazakii*. The name *Enterobacter sakazakii* was, and is, validly published and is available for those who might not agree with the proposed reclassification as the genus *Cronobacter*. A better and more precise term is “the *Enterobacter sakazakii* complex” which is equivalent to “*Cronobacter* species.”
(3) The “second proposed reclassification” was that of Iversen et al. who named and described *Cronobacter* with a total of 7 species/subspecies including *Cronobacter sakazakii*, the most important species.
(4) All strains originally classified as *Enterobacter sakazakii* need to be re-studied to see which *Cronobacter* species they belong to. Many will be *Cronobacter sakazakii*, but some will be other *Cronobacter* species.

For example, almost 40 years ago I isolated an organism from my dog’s water bowl and identified it as *Enterobacter sakazakii*. Today, this strain could be revived from a CDC freezer and retested with one or more sensitive identification methods now available. Its correct identification may be *Cronobacter sakazakii* or it may be one of the other *Cronobacter* species. When this is done a statement such as the following can be written:

“The organism that Farmer isolated in 1978 from his dog’s water bowl (CDC strain 1167-78) was originally identified as *E. sakazakii*. In 2015 it was removed from a CDC freezer and re-tested. The new identification was *Cronobacter dublinensis* subspecies lactaridi* based on the following criteria: PCR analysis, 16S r-RNA sequence analysis ….”

*Or whatever this revised identification turns out to be

The “Farmer” in “*Cronobacter sakazakii* (Farmer et al. 1980) Iversen et al. 2008” means that Farmer is “claiming credit” for this scientific finding. I saw a version of this statement in a rebuttal of a report that I had written. It was written by a well-known *Cronobacter* expert in a report he prepared for a legal case. He claimed that I was claiming credit for this organism, and seemed to imply that I did not have any right to do this. The term “*Cronobacter sakazakii* (Farmer et al. 1980) Iversen et al. 2008” is merely the correct way to cite this name under the rules of the *Bacteriological Code*. It has nothing to do with “claiming credit” as this expert incorrectly stated. His statement was based on a misunderstanding of the correct way to cite a species name when a new genus name is proposed. See the *Bacteriological Code*, and http://www.bacterio.net/cronobacter.html to verify the correctness of the above myth debunking.

*Question*: Are there other organisms in the family *Enterobacteriaceae* that can be confused with *Cronobacter* when they are being identified? Yes, strains in the “*Enterobacter cloacae* complex” can easily be confused. Strains typically identified as *Enterobacter cloacae* fall into at least five DNA-DNA hybridization groups depending on the operational definition used. The term “*Enterobacter cloacae* complex” might also include, or be confuses with, the organisms listed below (27):

*Enterobacter cloacae* DNA hybridization group 1
*Enterobacter cloacae* DNA hybridization group 2
*Enterobacter cloacae* DNA hybridization group 3
*Enterobacter cloacae* DNA hybridization group 4
*Enterobacter cloacae* DNA hybridization group 5
Enterobacter amnigenus
Enterobacter asburiae
Enterobacter cancerogenus
Enterobacter dissolvens
Enterobacter hormaechei
Enterobacter kobei
Enterobacter ludwigii
Enterobacter nimipressuralis
Enterobacter pyrinus
Enterobacter taylorae
Enteric Group 17
Others (depending on the operational definition used) such as some strains of *Erwinia, Brenneria, Pantoea, Pectobacterium, Enterobacter ludwigii*. For a more detailed analysis of this identification issue, see the discussions of Farmer et al. (27) in the *Manual of Clinical Microbiology*.

*Question*: Does the above discussions have anything to do with practical matters relating to *Cronobacter*? Yes. Every identification of a *Cronobacter* strain should be taken “with a grain of salt” or even better, the entire box of salt. The reader should critically examine the method(s) use in determining the identification. This is a particular problem if commercial biochemical identification methods (“commercial ID kits”) are used. They are not sensitive in distinguishing all of the organisms described in the preceding paragraphs.

*Questions*: I have seen the terms “*Enterobacter sakazakii* (s*ensu lato)”* and “*Enterobacter sakazakii* (*sensu stricto*)” – What exactly do they mean and why are these terms necessary? These terms are used to clarify the meaning of the words/terms “*Enterobacter sakazakii*” and “*Cronobacter sakazakii*.” They became necessary when the new genus *Cronobacter* was proposed in 2007. Below is a listing that should clarify this.

The organisms/terms below have the same definition and meaning and it is different from the names/organisms in the next grouping:

- *Enterobacter sakazakii* (s*ensu lato)*
- *Enterobacter sakazakii* (in a broad sense, those strains highly related to the type strain plus those less related but still now considered to be species of *Cronobacter*)
- *Enterobacter sakazakii* group
- *Enterobacter sakazakii* as defined by Farmer et al. (2)
- *Cronobacter* species

The organisms/terms below have the same definition and meaning and it is different from those in the previous grouping:

- *Enterobacter sakazakii* (s*ensu stricto)*
- *Enterobacter sakazakii* (in a strict sense, only those strains highly related to the type strain of *Enterobacter sakazakii*)
- *Cronobacter sakazakii* (only those strains highly related to the type strain of *Cronobacter sakazakii* and excluding all of the other *Cronobacter* species)

*Question*: What are some correct and incorrect usages of “*Enterobacter sakazakii”* from the pre-2007 literature?

*Correct*:

- In 1978, Farmer isolated a strain of *Enterobacter sakazakii* from his dog’s water bowl.
- In 1978, Farmer isolated a strain of *Enterobacter sakazakii* (s*ensu lato)* from his dog’s water bowl.
- In 1978, Farmer isolated a strain of the *Enterobacter sakazakii* group from his dog’s water bowl.
- In 1978 Farmer isolated a strain of the *Enterobacter sakazakii* complex from his dog’s water bowl.
- In 1978, Farmer isolated a strain of *Cronobacter* from his dog’s water bowl.
- In 1978, Farmer isolated a *Cronobacter* species from his dog’s water bowl.

*Incorrect*:

- In 1978, Farmer isolated a strain of *Cronobacter sakazakii* from his dog’s water bowl.

This last sentence would become correct if the strain isolated from his dog’s water bowl were re-studied and found to be *Cronobacter sakazakii*, rather than one of the other species of *Cronobacter*. Until this is done, it is best to refer to this organism as “*Cronobacter* species.”

## The Organisms and Their Properties

*Fact*: *Cronobacter* is a typical member of the family *Enterobacteriaceae* in many ways. This is documented in original 1980 paper by Farmer et al. and further described in dozens of subsequent publications.

*Fact*: One simple way to recognize a strain of *Cronobacter* is to take advantage of the very unusual way that colonies of *Cronobacter* grow on microbiological plating media. I described these colonies on page 576 (2) as follows: “… either dry or mucoid, crenated (notched or scalloped), and rubbery when touched with a loop (very little growth was removed and the colony snapped back when touched).” I noted that the colonies were very different from the typical colonies produced by other members of the family *Enterobacteriaceae* which are smooth, moist, and non-rubbery.

*Fact*: Another simple way to recognize a strain of *Cronobacter* is that most strains produce a bright yellow pigment. The original study found that 97% of the stains studied did this and that pigment production was much stronger at room 25°C than 36°C (2). However, the ability to produce yellow pigment was sometimes lost upon storage and subculture, which is true of other *Enterobacteriaceae* that produce distinct pigments.

*Fact*: Strains of *Cronobacter* can grow as individual cells, pairs of cells, and large clumps which contain hundreds/thousands of connected cells. This was shown in the original study (2).

*Fact*: Strains of *Cronobacter* can also grow as large sheets of cells called biofilms. This has been documented in many published studies.

*Fact*: Strains of *Cronobacter* have phenotypic properties and genetic properties that can be used as “strain typing methods” in microbial forensic analysis. The use of these genetic properties in epidemiological studies and causation analysis is illustrated in Table 1.

**Table 1:** **Example of how microbial forensic analysis can be used to “trace back” *Cronobacter* strains to a powdered formula factory – use of simple and reference (more complex) techniques to compare strains**.

Characteristic	*Cronobacter* strain 1	*Cronobacter* strain 2	*Cronobacter* strain 3	*Cronobacter* strain 4
**3 simple techniques**				
Biotype [of Ref. (2)]	Biotype 6, Indole+^a^	Biotype 6, Indole+^a^	Biotype 6, Indole+^a^	Biotype 1, Indole−^a^
Bionumber in the Vitek commercial test system	662475**1**67B^b^	662475**1**67B^b^	662475**1**67B^b^	662475**4**67B^b^
Antibiotic susceptibility pattern	Pattern 1 – sensitive to all	Pattern 1 – sensitive to all	Pattern 1 – sensitive to all	Pattern 6 – tetracycline resistant
**5 reference techniques**				
Plasmid profile	Pattern 1	Pattern 1	Pattern 1	Pattern 7a
PFGE – enzyme 1	Pattern 1	Pattern 1	Pattern 1	Pattern 14
PFGE – enzyme 2	Pattern 1	Pattern 1	Pattern 1	Pattern 19
MLVA	Pattern a	Pattern a	Pattern a	Pattern c
Multilocus sequence typing (MLST) of Joseph et al.	Type 4	Type 4	Type 4	Type 1
Ribotype	Type 2	Type 2	Type 2	Type 9
Final identification	*Cronobacter sakazakii* sequence type 4	*Cronobacter sakazakii* sequence type 4	*Cronobacter sakazakii* sequence type 4	*Cronobacter sakazakii* sequence type 1

*^a^Biotype 6, Indole+ is a very unusual biotype, but Biotype 1, Indole− is a very common biotype [see Table 3 of Ref. (2)]*.

*^b^Indole production is also one of the tests in this commercial identification system. Thus, *Cronobacter* strains that are indole positive have a different bionumber than strains that are indole negative (contrasting numbers are highlighted in bold type above)*.

*Conclusion: Although the two cases of neonatal meningitis are separated in both time and geography, they were both probably, to a high degree of scientific certainty, infected by the same strain of *Cronobacter* that can be traced back to a contaminated powdered formula made at factory A*.

*This following is a simulation based on several different investigations*.

*The purpose is to show by microbial forensic techniques that strains 1, 2, and 3 are a “microbial forensic match” and that strain 4 is a “microbial forensic mis-match” to strains 1–3*.

*Cronobacter strain 1: isolated from a neonatal meningitis case in a Georgia hospital in June of 2005*.

*Cronobacter strain 2: isolated from a neonatal meningitis case in a Maryland hospital in March of 2006*.

*Cronobacter strain 3: isolated during an FDA investigation in April, 2006 from a contaminated piece of production equipment (storage hamper for finished product) at factory A “dryer deluge”, “leakey nozzle in dryer”*.

*Cronobacter strain 4: isolated from a sink drain of the nursery at the Maryland hospital during an investigation of cause of the Maryland case’s meningitis*.

*Both cases ingested powdered formula made in factory A. They both ingested powdered formula for 1–6 days before they developed meningitis*.

*Facts*: Species/strains of *Cronobacter* have different pathogenic potentials for causing meningitis in infants and babies. *Cronobacter sakazakii* sequence type 4 (*C. sakazakii* ST 4) is extremely important as a cause of neonatal meningitis. Joseph and Forsythe (4) studied a collection of 41 *Cronobacter* strains and found that they could be defined in terms of different “sequence types.” They gave “sequence types designations” for these that went from 1 to 41 (ST1 – ST41) (see Table 1 in their paper). A summary of findings from the paper:

- Of the 20 *C. sakazakii* ST4 strains, ten were from neonates; seven were from infants; one was from a child and the source of the other was unknown.
- Seven of the *C. sakazakii* ST4 strains had been isolated from spinal fluid, two were from cases of necrotizing enterocolitis and one was from a case of bacteremia.
- Half (20 of 41) of the *C. sakazakii* strains were ST4, and 9 of 12 meningitis isolates were ST4.
- *C. sakazakii* ST4 appears to be a highly stable clone with a high propensity for neonatal meningitis.
- *C. sakazakii* ST4 appears to be a stable clone because strains have been isolated from 7 countries for > 50 years. The earliest non-clinical isolate was isolated in 1950 from a can of dried milk.

This illustrates the unique position of *C. sakazakii* ST4 in relation to other *Cronobacter* species and strains, and in relation to meningitis in neonates and babies. This is analogous to the unique position of *Escherichia coli* K1 as a cause of meningitis, which is in contrast to other species and strains in the genus *Escherichia*.

The importance of *C. sakazakii* ST4 is important in a critical analysis of the statement “*Cronobacter* is widely distributed in the environment.” This should be reframed to the question “*Cronobacter/E. sakazakii* strains have been found in several different kinds of environment specimens, but do any of these environmental strains have the capability of causing neonatal meningitis?”

## Environmental Stress, Injured Cells, and the “Viable but Non-Culturable” State (the “VNC” State)

*Facts*: *Enterobacteriaceae* strains including *Cronobacter* can become injured when they are subjected to stress such as heat and drying. Bacteria can respond to stress by entering a unique physiological state known as the “viable but non-culturable” (often abbreviated as the “VNC” state”). Many different studies indicate that pathogens in the family *Enterobacteriaceae* can enter this state. These include *Escherichia coli* O157:H7, *Salmonella*, *Shigella*, *Enterobacter cloacae*, *Klebsiella*, and many others. See the review by Oliver (28) for a complete listing and details.

*Question*: Can *Cronobacter* strains react to stress conditions and pass from a “viable and culturable state” to a “viable but non-culturable state?” Until recently *Cronobacter* had not been studied in regard to the viable but non-culturable state. Similarly, until recently laboratories had to resort to research procedures to look for this phenomenon. However, that there are now commercially available reagents and procedure for reviving, detecting and isolating strains of *Cronobacter* that are in the viable but non-culturable state.

*Observation 1:* At the International Conference on *Cronobacter* (*Enterobacter sakazakii*), Dublin, Ireland, January 22-23, 2009 the first data on the viable but non-culturable state for *Cronobacter/E. sakazakii* was presented. Dr. Angelica Lehner is one of the foremost *Cronobacter* researchers. She works at the Institute of Food Safety and Hygiene, University of Zurich, Zurich Switzerland and has done experiments using *E. sakazakii* strain E 601 (ATCC 29544), which is a non-capsule producer and *E. sakazakii* strain E 602 which produces capsules. She stated that **“Cronobacter seems to enter a viable but non-culturable state”** – see: http://crono09.tripod.com/lehner.pdf.

*Observation 2:* The commercial company Sigma Aldrich states: “Supplementing the pre-enrichment and enrichment broths with ferrioxamine E significantly improved the recovery of *Salmonella*, *Cronobacter* spp., *Staphylococcus aureus* and *Yersinia enterocolitica* from artificially or naturally contaminated foods [1–3]. A concentration of ferrioxamine E (available from Sigma, see Table 1) in the range of 5–200 ng/mL supports growth (see Table 2). … This leads to a reduced lag-phase in the medium and reactivates damaged bacteria. The ferrioxamine E is often used in Buffered Peptone Water the medium recommended by the ISO-Norms for *Enterobacteriacea* ….” (see Table 3)

**Table 2:** **Correlation between the number of *Cronobacter sakazakii* cells and colony forming units; must probable number values in a 100-g sample of a powdered formula product**.

Growth form of the *C. sakazakii* cells in the powder formula	Colony forming units (cfu) per 100 g	Most probable number (mpn) per 100 g	Actual number of cells per 100 g
Single cell	1	1	1
Pair of cells that are joined together	1	1	2
Clump of 100 cells	1	1	100
Clump of 10,000 cells^a^	1	1	10,000
Biofilm of 1,000,000 cells	1	1	1,000,000
Biofilm of 1,000,000,000 cells	1	1	1,000,000,000
Colony A in Figure 3b of Farmer et al. (2)^b^	1	1	1,000,000,000,000

*This table is a simulation of how different growth states of a C. sakazakii culture can affect conclusion about the degree of contamination of a powdered infant formula product. In the simulation above, one of the three 100 g samples of a powdered infant formula product was tested by the FDA MPN method and found positive for C. *sakazakii* contamination. Depending on the degree of clumping/biofilm formation strikingly different conclusion would be reached on the degree of contamination of the powdered product – contrast the results in columns 2 and 3 with column 4*.

*^a^A clump of cells such as this would be found at the bottom of the test tube shown in Figure 4, (right side, *E. sakazakii* strain 38) of Farmer et al. (2)*.

*^b^Assumptions: the colony weighs 1 g, and a single *E. sakazakii* cell weighs 1/1,000,000,000,000 of a gram*.

**Table 3:** **Recommended end concentration of Ferrioxamine E**.

Organisms	Nanogram per milliliter
Salmonella	75
Cronobacter spp. (*Enterobacter sakazakii*)	150
*Yersinia enterocolitica*	100

*See more at: http://www.sigmaaldrich.com/technical-documents/articles/microbiology-focus/viable-but-nonculturable.html*.

Table 4 lists a few ways to “revive” *Cronobacter* and other bacteria from the non-culturable state to the culturable state.

**Table 4:** **Commercial and non-commercial treatments and reagents that have been used to change *Cronobacter/E. sakazakii* and other *Enterobacteriaceae* from the viable but non-culturable state to the culturable state**.

Organism	Treatment	Reference
*Cronobacter/Enterobacter sakazakii*	Commercial Ferrioxamin E (FerriOx), 150 ng/mL	Sigma Aldrich Company
*Cronobacter/Enterobacter sakazakii*	*In vivo* passage in re-colonization plant model for seeds	Lopez (30)
*Campylobacter jejuni*	Intestinal passage in suckling mice (neonatal mouse model)	Oliver (28)
*Enterobacteriaceae* – various species	Numerous ways and conditions	Oliver (28)
*Escherichia coli*	Trihydroxamate siderophore ferrioxamine	Reissbrodt (31)
*Escherichia coli*	Commercial antioxidant Oxyrase	Reissbrodt (31)
*Escherichia coli*	“Enterobacterial autoinducer” (a heat-stable autoinducer of growth produced by enterobacterial species in response to norepinephrine)	Reissbrodt (31)
*Salmonella* serotypes	Commercial Ferrioxamin E (FerriOx), 75 ng/mL	Sigma Aldrich Company
*Salmonella* Typhimurium	Trihydroxamate siderophore ferrioxamine	Reissbrodt (31)
*Salmonella* Typhimurium	Commercial antioxidant Oxyrase	Reissbrodt (31)
*Salmonella* Typhimurium	“Enterobacterial autoinducer” (see above)	Reissbrodt (31)
*Yersinia enterocolitica*	Commercial Ferrioxamin E (FerriOx), 100 ng/mL	Sigma Aldrich Company

The existence of *Cronobacter* strains in the “viable but non-culturable state” is a possible explanation for why a sample of powdered infant formula or other product can be tested and found “negative for *Cronobacter* contamination” but is actually contaminated with *Cronobacter*. This explanation would be that the lack of a sensitive testing method resulted in a false negative test result.

However, further studies are needed to provide a definitive answer to the above question. If *Cronobacter* strains can go from a “viable and culturable state” to a “viable but non-culturable state” work is needed to establish “frequency of occurrence” in different types of environments, particularly in the production of powdered infant formula products. It is essential for companies that produce these products to evaluate their procedures and final product for *Cronobacter* contaminants that are in the viable but non-culturable state. I have seen no evidence that powdered infant formula manufacturers have set this up as a general microbiological procedure or have done a specific evaluation in its causation analysis of a specific case of *Cronobacter* meningitis. These should be done as safety procedure.

The reader is urged to follow the development of this topic with frequent literature and internet searches.

## Isolation, Identification, Typing Methods

*Facts*: Many different methods have been used to isolate and identify *Cronobacter*. Some isolation methods are good, but others give false negatives for the presence of *Cronobacter*. Each isolation method has its advantages and disadvantaged, and there is an extensive literature describing them. Many different methods have been used to identify *Cronobacter*. Some are good, but others give incorrect identifications. Each identification method has its advantages and disadvantaged, and there is an extensive literature describing them.

*Facts*: Not all strains identified as *Cronobacter* are really *Cronobacter*. Similarly, not all strains identified as, or referred to, as *Enterobacter sakazakii* are really *Enterobacter sakazakii*. These incorrect identifications are causing confusion in the literature.

*Fact*. The MPN method is a microbiological culturing method and is frequently used to determine the degree of *Cronobacter* contamination in a sample of powdered infant formula and in other foods. When the MPN method is used, the resulting report states the degree of contamination in terms of MPN per gram of the sample, or MPN per 10 g and/or MPN per 100 grams.

*Myth*: In the MPN method for *Cronobacter*, one colony forming unit (CFU) represents one viable cell of the *Cronobacter* contaminant. “Total viable counts” are often done by the MPN microbiological assay to determine contamination levels. This assay assumes that a positive result (positive tube) results from one cell of the contaminating bacterium. However, strains of *Cronobacter* can form large masses of adherent cells and can also form biofilms. Thus, a positive tube in the MPN assay may not have been caused by a single cell. This is illustrated in Table 2.

*Myth*: Two strains of *Cronobacter* that have the same PFGE pattern are the “same strain in a genetic/epidemiological sense.”

*Myth*: Two strains of *Cronobacter* that have different PFGE patterns cannot be the “same strain in a genetic/epidemiological sense.”

The reason for the two myths above is that there are too many variable in the PFGE laboratory technique to make precise statements such as these.

## Strain Preservation

*Fact*: There are many good methods to preserve strains of *Cronobacter* once they have been isolated and identified.

*Recommendation*: Investigators should permanently preserve each *Cronobacter* strain that isolate and identify in order to allow for future study.

Unfortunately, investigators often destroy or discard strains of *Cronobacter* that they have so carefully isolated, identified and studied. An employee of the powdered infant formula industry stated that in his opinion it is not useful to preserve *Cronobacter* strains isolated from powdered infant formula, raw ingredients or from the factory environment. Perhaps he was thinking in terms of possible legal liability rather than in terms of helping an investigation of the causal role of the powdered formula in a case of neonatal meningitis following ingestion of powdered formula made in his production facility.

## Human Infections and Their Epidemiology

*Fact*: *Cronobacter* is a cause of neonatal meningitis. This is a well-documented fact. *Cronobacter* is also a well-known cause of hospital outbreaks and sporadic cases both in the home and hospital.

*Myth*: *Cronobacter* causes diarrhea and necrotizing enterocolitis (NEC). It is true that *Cronobacter* has been isolated from cases of diarrhea and necrotizing enterocolitis. There is an important adage “association does not prove causation,” and this is true for these two human illnesses. Additional studies are needed based on well-established causation criteria.

*Recommendation*: Use the wording “*Cronobacter* has an association with diarrhea and necrotizing enterocolitis rather than “*Cronobacter* causes diarrhea and necrotizing enterocolitis.”

*Myth*: *Cronobacter* causes a wide variety of other human infections.

*Recommendations*: I would reword this to: “*Cronobacter* has been isolated from a wide variety of other human infections, but in the vast majority of these it was not proven that the organism was actually causing an infection.” This is the “infection vs. colonization” problem. Investigators should use serodiagnostic testing to determine if the case had an antibody response to the *Cronobacter* strain that was isolated.

*Myth*: The incubation period for human neonatal meningitis caused by *Cronobacter* is 3–4 days. It is true that in outbreak investigations symptoms developed as soon as 3–4 days after the initial ingestion of the implicated formula product. However, because of the many variables involved this observation does not prove, or even imply, that the incubation period was 3–4 days for the cases studied.

*Recommendation*: I would use this wording: “The incubation period for human neonatal meningitis caused by *Cronobacter* is unknown. Because of technical difficulties it will be very hard to determine the range and average incubation period.”

*Facts*: The infectious dose is unknown for human neonatal meningitis caused by *Cronobacter*. The infectious dose and incubation period are also unknown for all other infections caused by *Cronobacter*.

*Questions*: What does it mean when a *Cronobacter* strain is isolated from a sink drain in the home of an infant who has been diagnosed with *Cronobacter* meningitis and the *Cronobacter* strain found in the sink drain is a “molecular match” to the strain isolated from the infant? Does this prove or imply that the home environment is the source/cause of the infant’s *Cronobacter* meningitis? No, there are several possible explanations. The following is taken from public documents in an actual legal case which is based on investigations by government agencies including CDC:

There are several possible explanations for the origin of the *E. sakazakii* strain in the left sink drain at the house where the infant lived:

(1) The strain was present in one of the implicated batches of powdered formula made by company A, and it was in a “viable and culturable” state. The unused formula was poured into the sink. This may have occurred as early as October 29 or 30, the date the mother first fed the infant formula made from powder. One or more *E. sakazakii* cells in the discarded liquid adhered to the sink drain, probably adhering to a biofilm that was already present the drain. The *E. sakazakii* strain from the formula then colonized the sink drain, probably as a stable biofilm. The sink drain was later tested by CDC and the strain of *E. sakazakii* was isolated.
(2) The facts are the same as in number 1 above except *E. sakazakii* in the formula was in a “viable and non-culturable.” The favorable environment of the sink allowed *E. sakazakii* to be “revived” and transformed into a “viable and culturable” state. It was later isolated and identified at CDC.
(3) The facts are the same as in number 1 above except *E. sakazakii* was in the feces of the infant in the days before his *E. sakazakii* infection began, i.e., he had intestinal colonization with *E. sakazakii*. It was then transferred to the sink/sink drain by one of many possible mechanisms. One mechanism would be the hands of someone who came in contact with his feces and then used the sink.
(4) Something or someone introduced the *E. sakazakii* into the sink. From the sink drain it may have been transferred to the infant in some way and then caused his infection. This explanation was given by one or more defense experts.

*Fact*: Not all species/strains of *Cronobacter* have the same pathogenic potential. This was emphasized in the previous discussion about the importance of *Cronobacter sakazakii* sequence type 4 (*C. sakazakii* ST 4) in neonatal meningitis.

## Animal Infections, Animal Models of Disease

*Fact*: There are no known animal infectious diseases caused by *Cronobacter*. Strains of *Cronobacter* have been isolated for animals, but these have been in the absence a naturally occurring disease process.

*Fact*: Animal models such as the neonatal rat model have been used to study the infectious process and possible virulence factors of *Cronobacter*.

*Myth*: Results from animal models accurately predict and can be extrapolated to the human disease processes to quantify items such as infectious dose and incubation period. Although animal models have been helpful in the absence of human data they have many limitations, and the results should be viewed with caution (29).

## Environment

*Fact or myth?*: *Cronobacter* is widely distributed in the environment. This is myth or a fact depending on the definition of “widely distributed.” A safe way to avoid making this an unanswerable question is to stick to the facts and use precise and accurate wording, i.e., write precisely sentences and include references:

Strains of *Cronobacter sakazakii* have been isolated from the following sources: … (References).
Strains of *Cronobacter sakazakii* sequence type 4 have been isolated from the following sources: … (References).
Strains of *Cronobacter condomentii* have been isolated from the following sources: … (References).

## Powdered Infant Formula Industry, and the International Formula Council (IFC)

*Facts*: The powdered infant formula industry and the IFC have played an important role in the current status of powdered infant formula in the United States in the following areas: manufacture, marketing, labeling, warnings, and instructions for preparation and use. They have influenced these in many different ways. However, it is beyond the scope of this paper to discuss these in detail. Several were discussed previously and additional one are listed in Tables 5 and 6.

**Table 5:** **Uncertainties associated with assessing the public health risk from *Cronobacter***.

• Cases of infection that are missed because of inadequate microbiological methods for detection, isolation and/or identification
• Cases that are not reported to local, state and federal health agencies
• Cases that are identified as *Cronobacter* infection or colonization but are not because of mis-identification of the bacterium that was isolated from the clinical specimen
• Differentiation of infection vs. colonization
• Incorrect use of the term “*Cronobacter* infection” – in most instances the correct usage is “a clinical microbiology isolate of *Cronobacter* which was not further studied as to infection vs. colonization”
• Incubation period, infectious dose and strain infectivity in neonatal meningitis and other human infections
• Incorrect assumptions in causation analysis
• Possible role of throat colonization in neonatal meningitis as a means of multiple inoculation of the intestinal tract (as has been shown for *Yersinia enterocolitica* serotype O3)
• Inaccurate medical records – Example: a twin whose record said he was not fed powdered infant formula, but he probably was because of a three different identification errors (switches) of his records with those of his twin brother who was fed a powdered infant formula)
• Animal models for infectious dose and incubation period have many limitations when extrapolated to human infections
• Unknown importance environmental reservoirs
• The original source of the *Cronobacter* organism isolated in the “blender-associated cases”
• The original source of the *Cronobacter* organism in the “Nursery water cases”
• Importance of strains destroyed, rather than saved, by the powdered infant formula industry

**Table 6:** **Recommendations based on everything I have learned about *Cronobacter* over the last 40 years**.

**To microbiologists**
• Preserve all *Cronobacter* isolates for future study
• See previous paragraphs for the many others
**To government agencies**
• Make a *Cronobacter* infection a reportable disease as it’s the case for many other serious infectious diseases. In the United States the state on Minnesota has done this, but it is apparently be the only state that has established this reporting requirement
**To CDC – additional recommendations to add to your excellent recommendations for safely preparing infant formula**
**Powder examination steps**
• Examine the powder and other items used in formula preparation carefully before each feeding
• Examine the powder for insects, insect parts, or other foreign objects. One manufacturer had a serious contamination problem with beetles, beetle larvae, beetle parts. They had to recall millions of packages of this adulterated product^a^ (34). In my kitchen simulation experiments I sometimes saw flying and crawling insects in the areas around the sink that pose a possible contamination danger to the powdered formula or other items used in formula preparation
**Water safety steps**
• Always boil the water used in preparing formula, even if it is distilled or bottled water. Bacteria can contaminate water in numerous ways
**Safety steps for the scoop used to measure the amount of powdered formula**
• Insert a spoon or long forceps into boiling water for 1 min. This is a disinfection step to kill most germs that may be present
• Place the disinfected spoon or long forceps of a clean dish that has similarly been disinfected
• Insert the disinfected spoon or long forceps into the can of powdered infant formula to remove the measuring spoon
• Place the spoon in a small glass container that similarly been disinfected. Cover with aluminum foil to prevent contamination from germs in the air that can contaminate the spoon
• With your fingers touch only the handle of the scoop and remove it from its container
• Measure the correct amount of powder. Add water and mix as instructed
• Place the scoop back into the glass and cover it with foil
• By doing the above steps you greatly reduce the contamination of the powder with the scoop and your fingers
**To CDC – an additional recommendation**
• Do not use the word “infection” in “*Cronobacter* infection” unless infection has actually been documented
**To the powdered infant formula industry**
• Make a product that is free of contamination with *Cronobacter*, *Salmonella*, other pathogenic bacteria, other microorganisms, insects, insect parts, or other foreign objects
• Do much more intensive sampling and testing to detect and isolate *Cronobacter* and other pathogenic bacteria when you are testing for contamination of finished powdered formula, raw materials, the factory environment, and items supplied by contractors powdered formula
• Use sensitive and specific methods in the above testing that isolate and identify both viable and “viable but non-culturable” strains of *Cronobacter, Salmonella*, and other pathogenic bacteria
• Freeze or otherwise permanently preserved for future study all strains of *Cronobacter, Salmonella*, and other pathogenic bacteria isolated in the sampling and testing described above
• Notify FDA and when a strain of *Cronobacter, Salmonella*, or other pathogenic bacteria is isolated in any of the above
• Permanently preserve samples/cans (“library samples”) of powdered infant formula from each batch/lot made in the production facility. The reason for doing this is that additional testing is then possible to rule out or rule in the probable source when a case of infection by *Cronobacter, Salmonella*, or other pathogenic bacteria is reported following ingestion of the powder
• Implement CDC’s descriptions above (with improvements) for preparing infant formulas safety and add these to the label/instructions for each can of powdered infant formula
• Do not allow water to accumulate on the roof of the production facility! This was a documented problem at a Mead Johnson facility that produced powdered formula. The Structure Tech Company stated on its internet site: “Mead Johnson, a division of Bristol-Myers Squibb, was experiencing leakage conditions over their manufacturing operations, some of which were sterile environments.” The internet description above was apparently removed after this was damaging quotation was revealed in a legal case involving the Mead Johnson facility
**To FDA**
• Change from being an advocate of the powdered infant formula industry to being an advocate for public safety
• Require the powdered infant formula industry to implement the items listed above and those stated in previous paragraphs of this paper
• Provide documentation why you told the powdered infant formula industry it only needed to test one 333 gram sample of a lot of powdered formula when you own data from 2002 showed that this is an inadequate sample size, and that four 333 gram samples were needed to detect *Cronobacter* contamination
**To the International Formula Council**
• Do not distort facts in your role as an advocate for the powdered infant formula industry
• Do not make ridiculous statements such as “*Enterobacter sakazakii* is not a pathogen.” You lose all credibility when you act in an irresponsible manner in making a statement such as this
**To mothers and preparers of powdered infant formula in the home and hospital**
• Do breast feeding whenever possible
• When you cannot breast feed, use liquid formula rather than powdered infant formula
• When powdered infant formula is used, carefully read and follow CDC’s detailed description for preparing it more safely
• Add addtional safety steps listed above to further decrease injection risks

*^a^http://www.fda.gov/Safety/Recalls/ucm226885.htm*.

*Myth*: Manufacturer’s instructions for preparing powdered infant formula are complete and definitive in telling preparers how to avoid risks of infection by *Cronobacter* and other pathogens. There is there no universal standard or wording that commercial manufacturers of powdered infant formula are required to follow in this regard. The powdered infant formula industry and FDA have done a very poor job in providing complete information and instructions. Fortunately, CDC has taken the lead and has provided complete and specific instruction for preparing powdered infant formula which should greatly reduce the risk of *Cronobacter* infection and infection with other organisms. See: CDC’s information “*Cronobacter* Illness and Infant Formula” found at: http://www.cdc.gov/Features/Cronobacter/ and “*Cronobacter* Illness and Infant Formula” found at: http://www.cdc.gov/cronobacter/.

*Recommendations*: Based on published and unpublished information and on my personal experiences I have several recommendations for CDC to improve their excellent instructions/advice to reduce infection risks during preparation of powdered infant formula. These are listed in Table 6.

## Searching the Literature

The REFERENCE section below is a recommended starting point to learn more about *Cronobacter*. The key point to remember is that beginning in 2007 there was a transition in the usage of names from *Enterobacter sakazakii* to *Cronobacter*. For this reason, a literature search should use the following terms to capture all of the possibilities: “yellow-pigmented *Enterobacter cloacae*” “*Enterobacter sakazakii*” “*E. sakazakii*” “*Cronobacter sakazakii*” “*C. sakazakii*” “*sakazakii*” and “*Cronobacter*.” A very simple alternative is to use just two terms: “*sakazakii*” and “*Cronobacter*,” which I have found is very effective. Several papers are highly recommended (32, 33, 35–42).

## Author Note

This paper expands on many of the topics and ideas in my Keynote Lecture “Cronobacter (Enterobacter) sakazakii – Reflections on the First 50 Years; Challenges and Unresolved Issues for the Next 50,” that I presented at the 1st International Conference on Cronobacter (*Enterobacter sakazakii*), Dublin, Ireland, January 22, 2009 (43).

## Conflict of Interest Statement

The author declares that for over 10 years he has done expert witness work in legal cases that involved infants and babies who had ingested powdered formula products; became infected with *Cronobacter/E. sakazakii*; and suffered debilitating injury or death. This work included one or more of the following: detailed causation analysis, review of documents and procedures of the implicated manufacturer of the powdered formula product, written and oral reports, and testimony at deposition and trial. He was retained and received monetary compensation for this work regardless of the opinions and conclusions of his detailed causation analysis. Payments were from plaintiff attorney who represented the infant/baby who had suffered debilitating injury or death. The defendants in these cases were three U. S. manufacturers of the powdered formula product/products that were ingested by the injured infant or baby. Public records and non-sealed court documents describe some of the work/testimony described above. However, some work/testimony was “sealed by an order of the court,” or was covered by a “protective order” that I signed at the beginning of the cases. Thus, some of the above has been shielded from public view as a matter of law.

## Footnotes

^1^www.ucd.ie/crono09/
